# Opportunities for Successful Stabilization of Poor Glass-Forming Drugs: A Stability-Based Comparison of Mesoporous Silica Versus Hot Melt Extrusion Technologies

**DOI:** 10.3390/pharmaceutics11110577

**Published:** 2019-11-04

**Authors:** Felix Ditzinger, Daniel J. Price, Anita Nair, Johanna Becker-Baldus, Clemens Glaubitz, Jennifer B. Dressman, Christoph Saal, Martin Kuentz

**Affiliations:** 1 Department of Pharmaceutical Sciences, University of Basel, 4056 Basel, Switzerland; felix.ditzinger@fhnw.ch; 2 Institute of Pharma Technology, University of Applied Sciences and Arts Northwestern Switzerland, 4132 Muttenz, Switzerland; 3 Merck KGaA, 64293 Darmstadt, Germany; daniel-joseph.price@merckgroup.com (D.J.P.); anita.nair@merckgroup.com (A.N.); Christoph.Saal@merckgroup.com (C.S.); 4 Institute of Pharmaceutical Technology, Goethe University, 60438 Frankfurt, Germany; dressman@em.uni-frankfurt.de; 5 Institute for Biophysical Chemistry & Centre for Biomolecular Magnetic Resonance, Goethe University, 60438 Frankfurt, Germany; j.baldus@em.uni-frankfurt.de (J.B.-B.); Glaubitz@chemie.uni-frankfurt.de (C.G.)

**Keywords:** glass forming ability, hot melt extrusion, mesoporous silica, amorphous stability, supersaturation

## Abstract

Amorphous formulation technologies to improve oral absorption of poorly soluble active pharmaceutical ingredients (APIs) have become increasingly prevalent. Currently, polymer-based amorphous formulations manufactured by spray drying, hot melt extrusion (HME), or co-precipitation are most common. However, these technologies have challenges in terms of the successful stabilization of poor glass former compounds in the amorphous form. An alternative approach is mesoporous silica, which stabilizes APIs in non-crystalline form via molecular adsorption inside nano-scale pores. In line with these considerations, two poor glass formers, haloperidol and carbamazepine, were formulated as polymer-based solid dispersion via HME and with mesoporous silica, and their stability was compared under accelerated conditions. Changes were monitored over three months with respect to solid-state form and dissolution. The results were supported by solid-state nuclear magnetic resonance spectroscopy (SS-NMR) and scanning electron microscopy (SEM). It was demonstrated that mesoporous silica was more successful than HME in the stabilization of the selected poor glass formers. While both drugs remained non-crystalline during the study using mesoporous silica, polymer-based HME formulations showed recrystallization after one week. Thus, mesoporous silica represents an attractive technology to extend the formulation toolbox to poorly soluble poor glass formers.

## 1. Introduction

The increasing prevalence of poorly water-soluble drugs has driven the field of pharmaceutical technology to develop modern approaches for formulation development. A well-established technique is to formulate the drug in an amorphous form, which results in an increase in apparent solubility, dissolution performance, and subsequent oral bioavailability [1,2]. However, such an approach comes with difficulties related to thermodynamic instability of the amorphous state, which can lead to recrystallization and thus negation of the aforementioned formulation advantages [3].

To give guidance on the recrystallization tendency of drugs, Baird et al. developed a classification system based on a molecule’s “glass forming ability” (GFA). The GFA is related to recrystallization behavior from super cooled melts [4,5,6]. Three classes of substances were defined: class one (GFA-I) drugs recrystallize upon cooling from the molten state, class two (GFA-II) drugs recrystallize after a heating–cooling–heating cycle, and class three (GFA-III) drugs remain amorphous throughout the entire experiment. Although the classification was developed for undercooled melts, which can be directly related to hot melt extrusion (HME), it has proven to be accurate for solvent evaporation processes as well [7]. This is a particularly relevant consideration for mesoporous silica systems, given that drug loading is driven by solvent penetration into pores and subsequent evaporation [8].

GFA-I compounds, poor glass formers, are particularly prone to recrystallization in amorphous formulations [9]. One strategy to tackle this instability is to combine the drug with a polymer in an amorphous solid dispersion. A very common technique to manufacture amorphous solid dispersions is HME [10,11]. In this process, polymer and API are mixed in the molten state to form an extrusion strand, which is further processed into a solid dosage form, e.g., tablet or a capsule.

Another approach is to formulate GFA-I drugs with mesoporous silica. This is of particular interest due to the high stability of the amorphous API once it has been loaded into the porous network of the silica. This enhanced stability is related to nano-confinement in the meso-scale pores, which by definition range from 2–50 nm [12,13]. Stability is further improved with complementary pore-API interactions that lower the free energy of the system [14]. Muller and co-workers demonstrated amorphous stability at ambient and accelerated conditions for 30 different mesoporous silica formulations [15]. One key consideration for mesoporous silica formulations is the location of the API within the sample. For GFA-I compounds, it is essential that the loading process is carried out carefully, avoiding oversaturation of the silica, to ensure the drug is loaded within the pores and not on the outer surface. GFA-I compounds adsorbed on the outer surface are prone to rapid crystallization, which can be observed with techniques such as differential scanning calorimetry (DSC) and powder X-ray diffraction (PXRD). However, upon successful loading of the drug in the internal porous network, silica formulations can provide a viable alternative for drugs that fail to form a stable amorphous formulation in classical solid dispersions [16,17,18]. Although amorphous stability in mesoporous silica has been previously described, there has been no comparison on stability of poor glass formers in HME and mesoporous silica technology published to date. 

Certainly, solid-state stability is not the only important formulation consideration for poorly soluble drugs that are also poor glass formers. It is also essential to consider stability of the supersaturation generated upon dissolution. Indeed, recent work has demonstrated that, due to their high propensity for recrystallization, poor glass formers may also have issues with rapid onset of precipitation upon release, thus limiting their therapeutic potential [19]. This is in line with the well-established “Spring and Parachute” model [20], which identifies the need for additional excipients to sustain supersaturation of APIs in solution [21]. For polymer-based solid dispersions, the polymer may be able to meet both requirements: suspending the drug in an amorphous form and inhibiting precipitation from the supersaturated state. An example of such a polymer is polyvinyl alcohol that is interesting due to its low hygroscopicity and for which a special grade has been introduced recently for HME [22]. Unlike polymer-based solid dispersions, the ability of mesoporous silica to inhibit precipitation of the supersaturated API is limited. Therefore, it is often necessary to incorporate precipitation inhibitors into mesoporous silica formulations [23]. 

In this study, the amorphous stability of two model poor glass formers, haloperidol and carbamazepine, formulated as HME and with mesoporous silica, was investigated in line with ICH Q1 accelerated stability conditions [24] over three months. The stability was monitored by means of PXRD and underscored with DSC measurements of the samples before and at the end of the study. To the best of our knowledge, such a comparative study has not been reported previously. This stability comparison was complemented by non-sink release testing in biorelevant media [25,26] to monitor drug release and dissolution throughout the study [23,27]. It is important to note that non-sink dissolution was not used as a direct comparison between the two formulations, as the mesoporous formulations do not inhibit precipitation upon release. Rather, the release curves are demonstrative of the decrease in dissolution performance that can be observed upon solid-state transformation. Finally, solid-state nuclear magnetic resonance (SS-NMR) spectroscopy was applied to investigate any qualitative changes drug–polymer spectra in HME formulations over the duration of the stability study [28].

From a practical perspective, both drugs have no thermal instability, which avoids the risk of heat-induced degradation during the HME process [29,30]. The drug load selected for the technology comparison was the highest amount that enabled initial amorphous loading for both HME and mesoporous silica formulations, so that a direct comparison between techniques could be attained. 

It was hypothesized that mesoporous silica formulations of haloperidol and carbamazepine would show enhanced solid-state stability over time compared to solid dispersion obtained from HME.

## 2. Materials and Methods

### 2.1. Materials

Haloperidol, carbamazepine, HPLC grade acetonitrile, and HPLC grade methanol were purchased from MilliporeSigma (Darmstadt, Germany). Parteck MXP^®^ (PVA) and Parteck SLC^®^ were kindly provided by Merck KGaA (Darmstadt, Germany). FaSSGF/FaSSIF/FeSSIF powder to make biorelevant dissolution medium, Fasted Simulated Intestinal Fluid (FaSSIF), was obtained from Biorelevant (Biorelevant.com, London, UK).

### 2.2. Methods

#### 2.2.1. Thermodynamic Solubility Determination

FaSSIF was prepared by weighing 45 mg of FaSSGF/FaSSIF/FeSSIF powder into 45 mL of phosphate buffer (pH 6.5) [31]. API (2–3 mg) was accurately weighed into a Uniprep^®^ syringeless filter (5 mL; 0.45 µm). 2 mL of FaSSIF was added and the samples were agitated at 450 rpm for 24 h at 37 °C. The pH was checked at 7 h and adjusted with 0.1 N NaOH or 0.1 N HCl, if a deviation greater than ±0.05 pH units was observed. The final pH was also recorded after 24 h. 

Samples were filtered into the inner chamber of the Uniprep through the built-in PTFE 0.45 µm Whatman filter after 24 h. Filtrates were immediately diluted with acetonitrile and water (1:4 *v*/*v*) to avoid precipitation from the saturated solution. Samples were analyzed with UHPLC (Thermo Dionex Ultimate 3000, Thermo Fisher, Waltham, MA, USA) to determine the API concentration. API concentration was determined based on a standard calibration curve of nine standard concentrations (50, 30, 10, 5, 3, 1, 0.5, 0.3, 0.1 µg/mL). Three control samples of known concentrations (30, 3, 0.3 µg/mL) were prepared and used to check the robustness of the calibration curve. The determination was carried out in duplicate. 

#### 2.2.2. Ultra-High Performance Liquid Chromatography (UHPLC)

UHPLC analysis was performed using a Thermo Dionex Ultimate 3000 (Thermo Fisher, Waltham, MA, USA) equipped with a diode array detector at 282 nm for carbamazepine and 247 nm for haloperidol (Thermo Fisher, Waltham, MA, USA). The separation was achieved on an Acquity UPLC BEH column C8 (2.1 × 50 mm, 1.7 µm, Waters, Milford, MA, USA). The mobile phases A and B consisted of water:formic acid 999:1 (*v*/*v*) and acetonitrile:formic acid 999:1 (*v*/*v*), respectively. Gradient and flow rate is shown in Table 1. System management, data acquisition and processing were based on the Chromeleon™ software package, version 7.2 (Thermo Fisher, Waltham, MA, USA).

#### 2.2.3. Preparation of Hot Melt Extrudates

PVA was selected as an optimal polymer for hot-melt extrusion. This was based on three factors: (1) the grade of PVA was specifically designed for optimal HME due to particle size distribution and viscosity, (2) consideration of partial solubility parameters for both drugs, and PVA (3) low hygroscopicity of PVA to reduce water uptake in of the extrudates. 

Binary mixtures of polyvinyl alcohol (PVA, Parteck MXP ^®^) and drug at various drug loadings were mixed in a mortar and extruded on a ZE9 ECO twin-screw extruder by ThreeTec (Birren, Switzerland) with 9 mm diameter and 180 mm length co-rotating screws. A screw speed of 80 rpm was applied at a temperature of 190 °C through all three heating zones, which is in accordance with recommendation by the polymer manufacturer [32]. After extrusion, the extrudates were ground in a mortar, and the fraction retained between mesh sizes 150 and 425 µm was retained for use in the study. The final extruded mixtures were cooled to room temperature and stored in falcon tubes. Mixing feasibility of the selected polymer for both drugs was verified by the Hansen solubility parameters [33,34], which were calculated using the quantitative structure property relationship (QSPR) method of the COSMOquick software (COSMOlogic, Leverkusen, Germany, Version 1.6) [35,36]. For the investigation of the formulations, a 7.5% (*w*/*w*) drug loading of haloperidol and 20% (*w*/*w*) was used for carbamazepine. This was selected on the basis of the highest drug load that was initially successful for both formulation technologies, and was the result of a formulation screening.

#### 2.2.4. Preparation of API-Loaded Silica Formulations

Mesoporous silica formulations were prepared using the incipient wetness method [13]. API (3 g) was dissolved in acetone (300 mL; 10 mg/mL), which was added drop-wise at a rate of 0.5 mL/min to Parteck^®^ SLC mesoporous silica (7 g), under constant stirring and heating at 60 °C. After complete addition of the concentrated API solution, the samples were dried overnight in a vacuum oven at 60 °C to ensure complete removal of the solvent. The formulations were prepared at a drug loading of 7.5% (*w*/*w*) for haloperidol and 20% (*w*/*w*) for carbamazepine.

#### 2.2.5. Storage of Samples for Stability Studies

For storage of the samples in the stability study, each of the formulations was placed in a separate glass jar with a secure lid. A separate beaker containing saturated sodium chloride solution, also placed in the beaker, ensured a constant relative humidity of 75% in the surrounding environment [37]. This enclosed system was then placed in a stability cabinet set to 40 °C to obtain storage conditions in accordance with ICH Q1.

#### 2.2.6. Powder X-Ray Diffraction (PXRD)

Samples were prepared between X-ray amorphous films and measured in transmission mode using Cu-Kα1-radiation and a Stoe StadiP 611 KL diffractometer (STOE & Cie GmbH, Darmstadt, Germany) in transmission mode equipped with Dectris Mythen1K PSD (DECTRIS Ltd., Baden-Daettwil, Switzerland). The measurements were evaluated with the software WinXPow 3.03 by Stoe (STOE & Cie GmbH, Darmstadt, Germany), the ICDD PDF-4+ 2014 Database (ICDD, Newtown Square, PA, USA), and Igor Pro Version 6.34 (Wavemetrics Inc., Lake Oswego, OR, USA) Angular range: 1–40° 2θ; PSD-step width: 2° 2θ; angular resolution: 0.015° 2θ measurement time: 15 s/step, 0.25 h overall.

#### 2.2.7. Non-Sink-Mini-Dissolution in FaSSIF

The equivalent of 5 mg API of extrudate or API-loaded silica was weighed into a glass vial. Five milliliters of FaSSIF was added. The vials were agitated at 37 °C and 450 rpm in a shaker (IKA-Werke GmbH & CO. KG, Staufen, Germany) for 120 min. Samples of 0.3 mL were taken at 2, 15, 60, 90, and 120 min, filtered (0.45 PTFE Whatman filters), diluted with acetonitrile and water, and analyzed by UHPLC. The mini-dissolution trials were conducted in duplicate for all samples.

#### 2.2.8. Scanning Electron Microscopy (SEM)

Samples were prepared on carbon tape and imaged using a TM3000 Tabletop Scanning Electron Microscope (Hitachi, Tokyo, Japan), tungsten source, using low vacuum and accelerating voltage of 5 and 15 kV. A 4-Quadrant BSE detector was used, and imaging was at a magnification between 15× and 30,000×.

#### 2.2.9. Differential Scanning Calorimetry (DSC)

Samples were assessed by differential scanning calorimetry on a DSC 3 (Mettler Toledo, Greifensee, Switzerland). An amount of 5 to 9 mg sample was placed in a 40 μL aluminum pan with a pierced lid. A heating rate of 10 °C/min from 20 to 200 °C was applied under nitrogen purging at 200 mL/min. The thermograms were analyzed with the STARe Evaluation-Software Version 16 (Mettler Toledo, Greifensee, Switzerland).

#### 2.2.10. Solid-State Nuclear Magnetic Resonance (SS-NMR) Spectroscopy

SS-NMR experiments were conducted with magic-angle-sample (MAS) spinning using a Bruker 4 mm MAS HXY probe in double resonance mode with a Bruker Avance I 600 MHz wide bore NMR spectrometer (Bruker, Rheinstetten, Germany) with a 4 mm rotor. The readout on the probe thermocouple was set to 290 K. The sample spinning frequency was set to 10 kHz. All spectra were recorded with ^1^H-^13^C-cross polarization (CP) using a contact time of 1 ms. 100 kHz high power proton decoupling following the SPINAL64 scheme was applied during acquisition. The recycle delay was 3 s. The spectra were indirectly referenced to 4,4-dimethyl-4-silapentane-1-sulfonic acid (DSS) via the CH_2_ signal of Adamantane at 40.49 ppm.

## 3. Results

### 3.1. Macro- and Microscopic Changes

**Figure 1:**
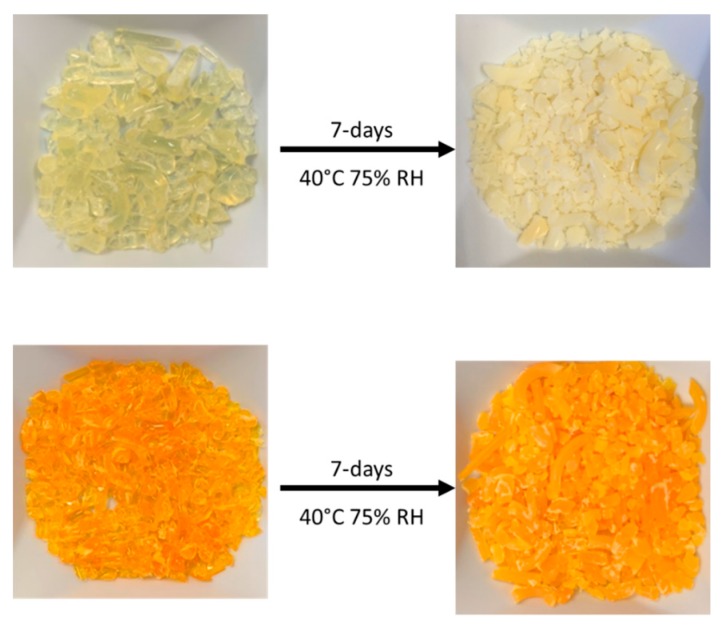
Haloperidol (top) and carbamazepine (bottom) hot melt extrusion (HME) before (left) and after (right) 7 days accelerated stability conditions as specified in the materials and methods section.

Qualitative macroscopic differences were observed between the fresh and one-week stressed samples of the hot-melt extrudates (Figure 1). Extrudates of carbamazepine and haloperidol were transparent immediately after manufacturing. This indicates the presence of molecularly dispersed API throughout the polymer in the amorphous form [11]. However, after only 7 days exposure to 40 °C and 75% RH, both extrudates became opaque, indicating phase separation in the formulations [38]. This was in contrast to mesoporous silica formulations, in which no macroscopic differences were observed between the fresh and one-week stressed samples. Indeed, the appearance of mesoporous silica formulations remained consistent over the duration of the 3 month study.

**Figure 2:**
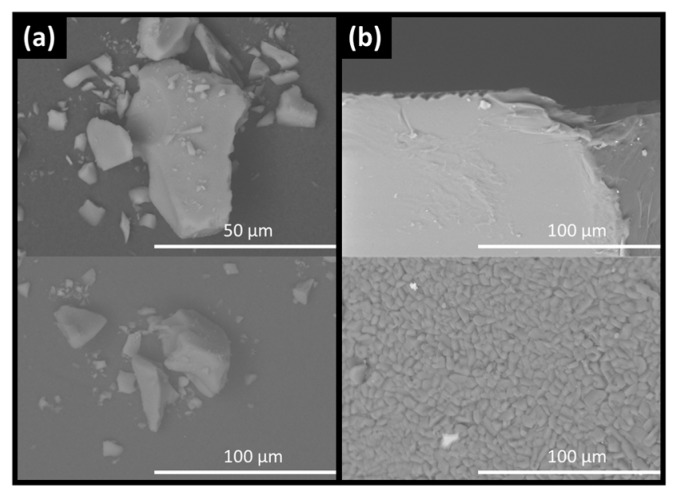
SEM images for carbamazepine loaded silica (**a**) and HME (**b**) showing particle size and morphology at 0 days (top) and 90 days stability (bottom) as specified in the materials and methods section.

**Figure 3:**
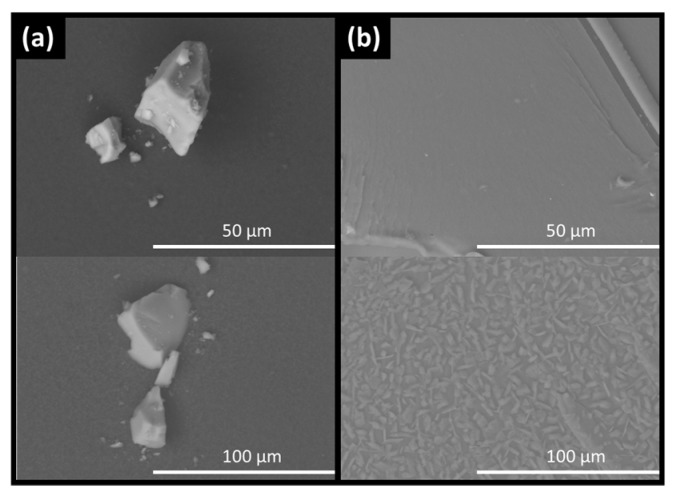
SEM images for haloperidol loaded silica (**a**) and HME (**b**) showing particle size and morphology at 0 days (top) and 90 days stability (bottom) as specified in the materials and methods section.

Changes in the extrudate samples over time were also observed on a microscopic level in the SEM images. In freshly prepared formulations, the extrudates showed no heterogeneity but at the end of the stability study, after 90 days, phase separation and recrystallization were observed. API-loaded silica formulations, however, did not exhibit qualitative changes under either visual inspection or by SEM (Figure 2; Figure 3).

### 3.2. Solid-State Stability of the Amorphous Form

**Figure 4:**
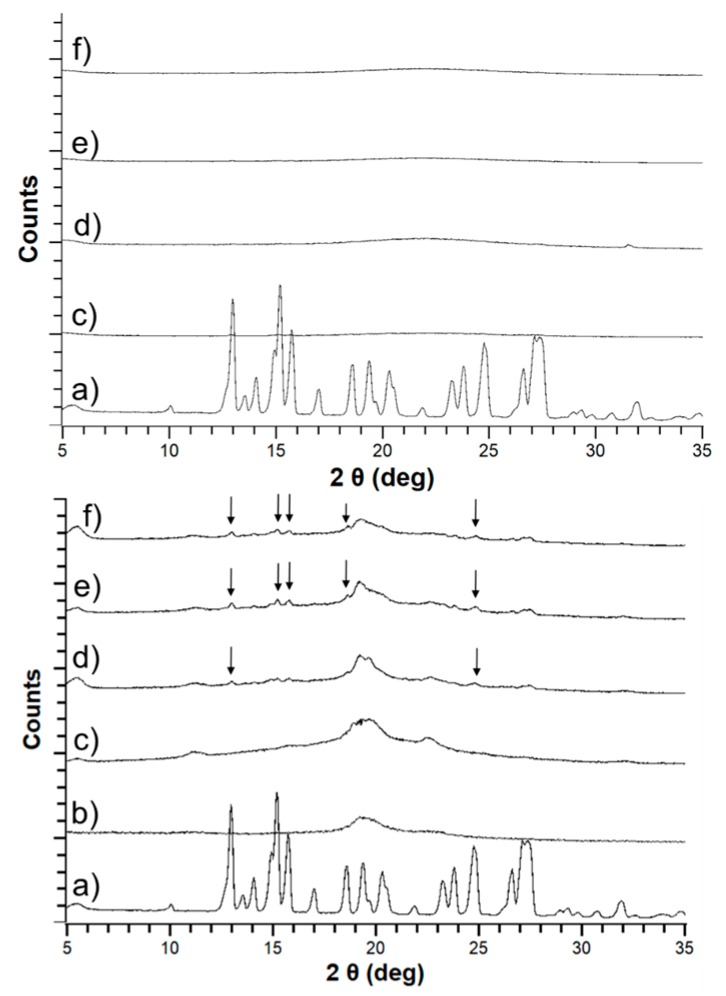
Powder X-ray (PXRD) patterns for carbamazepine loaded silica (top) and carbamazepine HME (bottom) showing crystalline carbamazepine (a), pure Parteck MXP^®^ (PVA) (b), unstressed carbamazepine formulation (c) and stressed carbamazepine formulations at 30 (d), 60 (e), and 90 (f) days. The arrows indicate crystalline peaks in the diffractograms.

**Figure 5:**
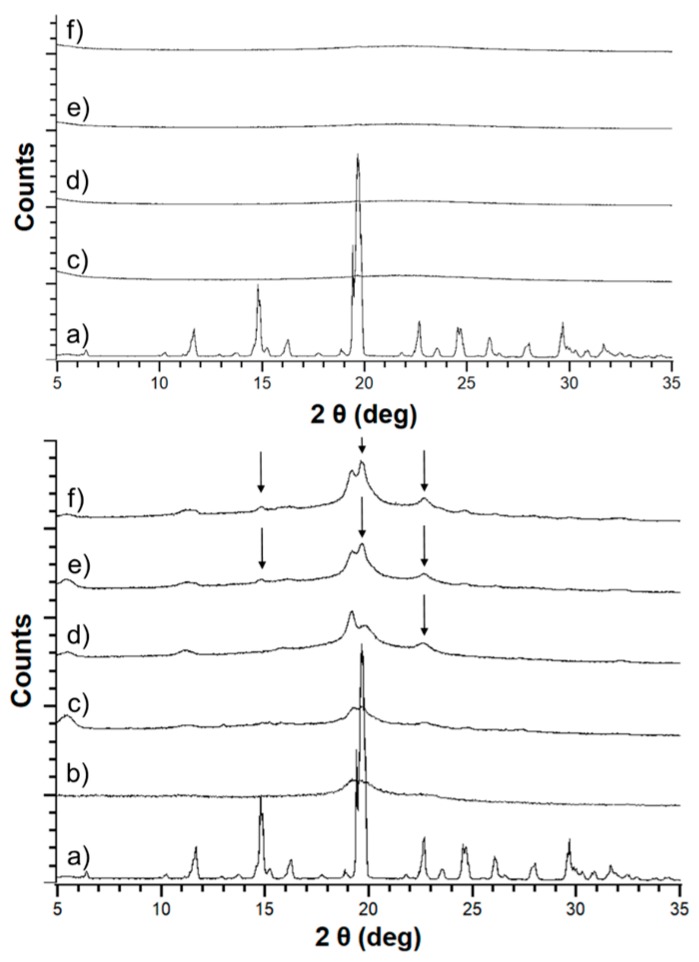
PXRD patterns for haloperidol loaded silica (top) and haloperidol HME (bottom) showing crystalline haloperidol (a), pure PVA (b), unstressed haloperidol formulation (c) and stressed haloperidol formulations at 30 (d), 60 (e), and 90 (f) days. The arrows indicate crystalline peaks in the diffractograms.

Both haloperidol and carbamazepine were crystalline before formulation with either HME or mesoporous silica. The outcome of the empirical loading approach is shown in Table 2. For mesoporous silica, both APIs were successfully stabilized in the amorphous form at an initial concentration of 30% (*w*/*w*). However, HME was only successful in stabilizing amorphous API for carbamazepine at 20% (*w*/*w*) and haloperidol at 7.5% (*w*/*w*) (data not shown). At higher concentrations, the extrudates were crystalline upon cooling. Therefore, for the comparative accelerated stability study of the formulations a drug load of 20% (*w*/*w*) carbamazepine and 7.5% (*w*/*w*) haloperidol was selected for both the mesoporous silica and HME based solid dispersions. PXRD indicated that the initial form in both formulations was amorphous (Figure 4; Figure 5).

Differences between silica-based formulations and PVA extrudates were apparent after one month of storage at elevated temperatures, with both HME formulations showing development of crystallinity. The crystalline percentage increased month by month over the duration of the study (Figure 4; Figure 5). 

Conversely, both API-loaded mesoporous silica formulations remained amorphous for the duration of the three-month stability study, with no evidence of crystallinity in the PXRD patterns (Figure 4; Figure 5). 

These findings were underscored by the absence of melting endotherms in the DSC thermograms of the silica-based formulations after 90 days [39]. By contrast, melting peaks were observed in both samples of the extruded formulations after 90 days, indicating the presence of drug crystallinity (data not shown).

**Figure 6:**
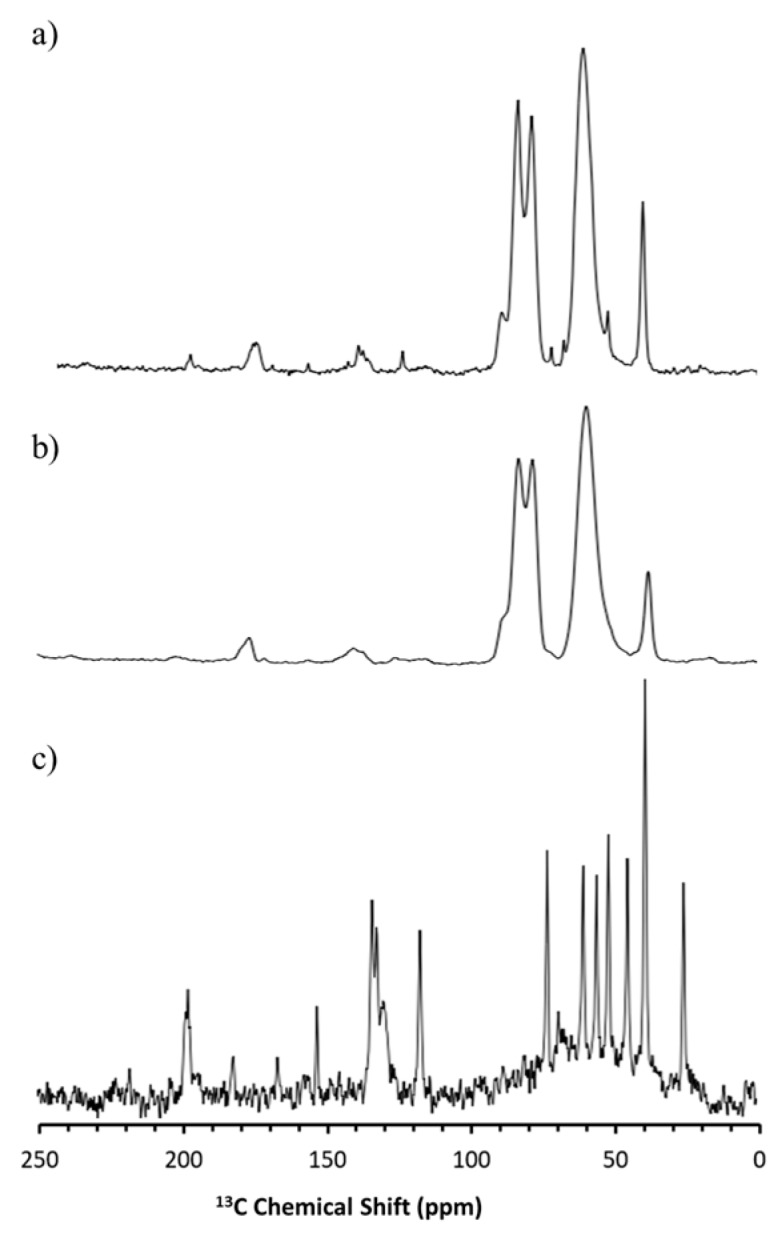
^13^C Solid-state nuclear magnetic resonance spectroscopy (SS-NMR) spectra for crystalline haloperidol (**c**), HME formulation at 0 days (**b**) and 90 days (**a**).

Although there were no pronounced drug–polymer interactions detectable in the SS-NMR spectroscopy for carbamazepine and haloperidol HME, it was possible to observe qualitative differences before and after storage of the samples at 45 °C/70% RH. Specifically, the freshly prepared samples had broad peaks in the spectra, related to the amorphous state of the sample. By contrast, an increased fine structure observed in the NMR-spectra at the end of the study indicated an increase in crystallinity. This was especially pronounced for haloperidol, with the stressed sample exhibiting peaks corresponding to the crystalline pure drug (Figure 6c) at 118, 153, and 200 ppm in Figure 6a.

**Figure 7:**
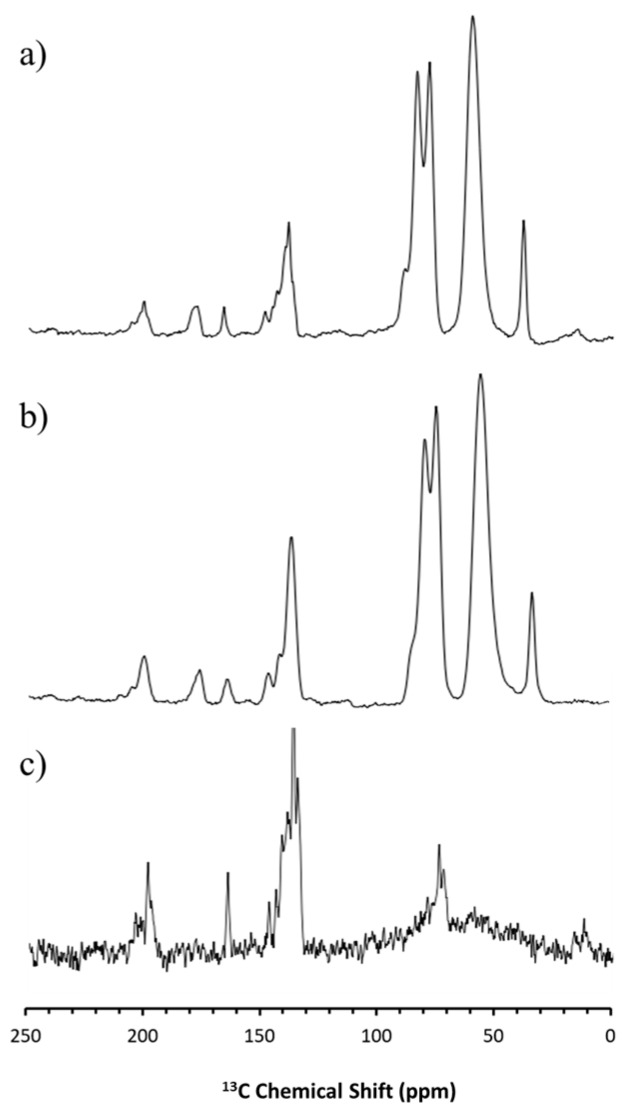
^13^C SS-NMR spectra for crystalline carbamazepine (**c**) and carbamazepine HME formulation at 0 days (**b**) and 90 days (**a**).

For carbamazepine, the change was more subtle, because of overlapping peaks. However, it was obvious that the stressed sample exhibited crystalline peaks that were not observed in the freshly prepared samples e.g., at 131 ppm, as shown in Figure 7. The presence of sharper peaks in the stressed samples underscores the recrystallization in the formulations suggested by the X-ray diffraction data.

### 3.3. Stability of the Supersaturated State in FaSSIF

The thermodynamic solubility of haloperidol and carbamazepine in FaSSIF was measured to be 259 (±1) µg/mL and 203 (±2) µg/mL. Accordingly, the crystalline APIs showed a dissolution profile approaching these values over the course of the FaSSIF dissolution experiment. 

Although both drugs have some solubility in FaSSIF, the dissolution was enhanced by both the mesoporous silica and HME formulations. For carbamazepine, a maximum supersaturation of 1.8 and 1.5-fold was generated for the silica and HME formulations, respectively. For haloperidol, a maximum supersaturation of about 2.0-fold was generated for both silica and HME. 

The PVA in the HME formulation was able to sustain supersaturated concentrations for both APIs by inhibiting precipitation from aqueous medium. Mesoporous silica, on the other hand, was unable to inhibit drug precipitation from the supersaturated state and therefore, precipitation was observed for both APIs, with concentrations returning to the thermodynamic solubility. For mesoporous silica formulations, further development of the formulation would include a screening and a selection of a precipitation inhibitor to include in the formulation. The precipitation inhibitor would prevent the precipitation of the supersaturated API and could subsequently enhance oral absorption. However, although important, the precipitation inhibitor in a mesoporous silica formulation is not expected to impact on the solid-state stabilization of the API in the amorphous form. This is due to the fact that precipitation inhibitors are simply blended with the drug-loaded silica when the drug is already loaded onto the porous silica and stabilized in the solid state. Therefore, as the focus of this study was on the innate stabilization potential of mesoporous silica using poor glass formers, incorporation of a precipitation inhibitor was out of scope. 

**Figure 8:**
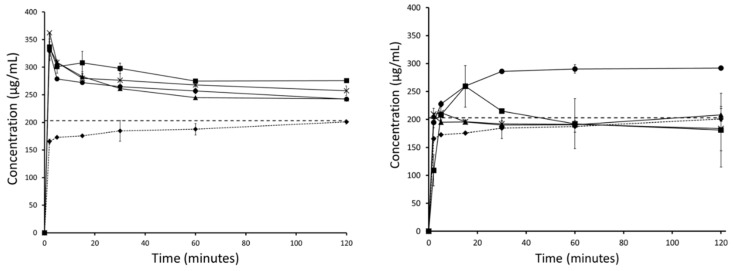
Fasted Simulated Intestinal Fluid (FaSSIF) mini-dissolution curves for carbamazepine loaded silica (left) and carbamazepine HME formulation (right) showing crystalline carbamazepine (♦), unstressed carbamazepine formulation (●), and stressed carbamazepine formulations at 30 (▪), 60 (X), and 90 (▲) days.

**Figure 9:**
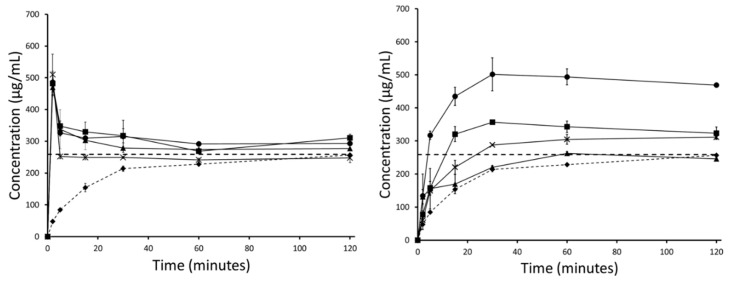
FaSSIF mini-dissolution curves for haloperidol loaded silica (left) and haloperidol HME formulation (right) showing crystalline haloperidol (♦), unstressed haloperidol formulation (●), and stressed haloperidol formulations at 30 (▪), 60 (X), and 90 (▲) days.

For both APIs, the dissolution profiles from mesoporous silica formulations were comparable throughout the duration of the entire accelerated stability study. Particularly notable was that the degree of supersaturation, or ‘spring’, remained consistent over the whole stability study (Figure 8; Figure 9). For HME formulations, the curves showed a decrease in supersaturation in each successive month of the stability study. After 30 days, the HME formulation containing carbamazepine was still able to generate supersaturation, but the profile was no longer stable; the carbamazepine concentration returned to the thermodynamic solubility within 60 min. This difference between fresh and 30 day carbamazepine samples was indicative of the presence of seed crystals in the formulations. Such seeds foster crystallization in the formulation as well as in solution to most likely override the inhibition of precipitation by the polymer [40]. Furthermore, the release performance of carbamazepine HME declined even further at 60 and 90 days. By 90 days, no supersaturation was observed at any measured time point during the experiment, and the dissolution curve resembled that of crystalline carbamazepine more closely (Figure 8).

Similar reductions in dissolution performance with storage were observed for the HME formulation of haloperidol. Although the dissolution performance of the haloperidol HME did not decline as quickly as that of the carbamazepine HME, its dissolution profile also resembled that of the crystalline API after 90 days (Figure 9). 

## 4. Discussion

Development of amorphous solid dispersions requires a thorough understanding of the factors that influence stability in the amorphous form. One such factor is the GFA. According to the classification system proposed by Baird et al. [4], GFA-I drugs are especially challenging when developing amorphous formulations, due to the high propensity for recrystallization of such compounds. It is essential to demonstrate good physical stability in amorphous formulations to ensure that recrystallization does not occur over time, as this would reduce the shelf life of the product [41]. As crystallization is based on the stochastic nucleation process, a lack of physical stability could lead to variable product quality among batches. Any initial crystallization in the batch, or differences in the rate of crystallization, could lead to out-of-specification results based on insufficient drug product performance, for example in dissolution testing. Variability among batches would thus be problematic in terms of meeting regulatory and commercial requirements. Herein, it has been demonstrated that mesoporous silica can be used to successfully stabilize compounds of poor amorphous stability that are unsuitable for formulation in standard polymer-based amorphous solid dispersions.

Initial formulation development demonstrated the potential impact that such a poor amorphous stability could have on the viability of a formulation. For both HME formulations, significantly lower percentage drug loads were attainable in the initial formulation development. This is a crucial topic for the development of amorphous formulations. Taking the example of haloperidol, it was only possible to stabilize 7.5% (*w*/*w*) of the API in the amorphous form in the HME. Assuming a theoretical dose of 200 mg, one would require a tablet of approximately 2.6 g in weight to incorporate the entire dose in a single dosage form. Furthermore, this represents a very conservative estimation, as the actual API content would likely be reduced further to 3.75% when one considers that 50% of the tablet may consist of fillers, binders, glidants, and disintegrants. Ultimately, a low drug loading would be a substantial risk to the viability of the formulation, and could result in failure of the project. Mesoporous silica, however, was successful in stabilizing more reasonable drug loads in non-crystalline form.

Furthermore, the successful low drug loading HME formulations developed for carbamazepine and haloperidol were observed to be unstable during the ICH Q1 stability study (Figure 4; Figure 5). Instability in polymeric amorphous solid dispersions can be linked with increasing temperature and humidity. As temperature or water content in amorphous formulations increases, mobility of the drug within the polymer dispersion increases. Mehta and co-workers demonstrated this effect on model amorphous solid dispersions (ASDs). In their study, an increase in molecular mobility of all APIs in the polymer ASDs led to a decrease in recrystallization time [41,42]. As both studies by Mehta and colleagues investigated the physical stability of good to moderate glass formers (GFA-II/III), it is likely that the effect of moisture and temperature on increasing molecular mobility and subsequent physical instability would be even greater for GFA-I compounds. The observed instability of these poor glass formers is even more remarkable when one considers that, of the available polymers for HME, PVA has a substantially low hygroscopicity. This low hygroscopicity would have a stabilizing effect on the formulation due to a reduction in the uptake of water upon storage at humid conditions. However, this beneficial characteristic of PVA was not enough to prevent the poor glass formers from recrystallizing in the extrudates [32]. 

For mesoporous silica, however, molecular mobility is greatly reduced regardless of moisture or temperature. Brás and colleagues demonstrated that adsorption and nano-confinement of ibuprofen onto mesoporous silica resulted in a significant reduction of all known types of molecular mobility [43]. Most interestingly, the Johari–Goldstein β relaxation, a type of molecular mobility associated with recrystallization, was reduced. This was a crucial observation, as it has been shown that increased Johari–Goldstein relaxation is related to physical instability of the amorphous form [42,43]. Similar to the work by Mehta and colleagues, Brás et al. focused on good glass formers (GFA-III). Additional work demonstrated that a reduction in molecular mobility leads to successful stabilization of the very poor glass former menthol (GFA-I), which has a glass transition temperature of −54.3 °C [44]. This stabilization was related to a decrease in molecular mobility of both α (free transitional mobility in space) and the aforementioned Johari–Goldstein β relaxations. Furthermore, a new type of molecular mobility, the S-type, was observed. S-type refers to mobility of a hindered molecule that is nano-confined within a single pore, and is much slower than standard molecular mobility events [44]. Based on these findings as well as those of our study (Figure 4; Figure 5), mesoporous silica may be a suitable way forward to stabilizing GFA-I glass formers under accelerated conditions.

There are only a handful of known GFA-I compounds that are also BCS II compounds and which would thus benefit from the apparent solubility increase of the amorphous form. In a recent review, Kawakami provided an overview of pharmaceutical compounds according to GFA classes [45]. Of the GFA-I compounds in the database only 29% were determined to be BCS II/IV, which is far lower than the commonly reported percentage of commercial compounds that fall into the poor solubility category (60%) [8]. Hence, there appears to be a disconnect between the prevalence of compounds with poor solubility and the occurrence of poor glass formers on the market. This could be related to the difficulty in formulating such compounds, and the reduction in formulation performance related to physical instability. 

For the two model BCS II drugs selected in this study, clear differences were observed in the non-sink release profiles of loaded silica and HME formulations. As expected from the literature, silica alone was not able to sustain supersaturated concentrations of API in solution resulting in precipitation [23]. Conversely, in the HME formulations, the API is sustained in solution by the polymer itself, which can function not only as a matrix polymer but also as a precipitation inhibitor during drug release. However, it was observed that dissolution of API loaded silica formulations remained consistent throughout the 3 month study (Figure 8; Figure 9), whereas the kinetic release of HME formulations tended towards crystalline drug solubility (Figure 8; Figure 9). Here, we see the effect of phase separation and recrystallization on the dissolution performance of amorphous solid dispersions, with the presence of a crystalline phase reducing the achievable supersaturation and decreasing the dissolution performance of the compound [45]. Interestingly, both HME formulations retained some supersaturation after the first month of the stability study, indicating that full conversion from amorphous to crystalline had not yet occurred (Figure 8; Figure 9). However, the supersaturated solutions generated by the carbamazepine HME were less stable than those generated by the haloperidol HME, and precipitation occurred (Figure 8). This was related to the presence of seed crystals in the formulation, which sped up the rate of nucleation and reduced the ability of the polymer to prevent precipitation. Patel and co-workers demonstrated that a small amount of crystalline indomethacin significantly increased its recrystallization from the supersaturated state, even in the presence of precipitation inhibitors [40]. The present results support the view that GFA-I compounds may not be good candidates for formulation in polymeric amorphous solid dispersions, such as hot-melt extrudates, which have been investigated here. However, this is also expected by other formulations based on polymeric amorphous dispersions, e.g., spray dried dispersions or co-precipitates. Mesoporous silica, on the other hand, is an attractive formulation option for poorly soluble glass formers, generating consistent and supersaturated dissolution profiles.

## 5. Conclusions

The increasing prevalence of poorly soluble BCS II drug candidates in pharmaceutical development remains a challenging issue. Although polymer-based stabilization of the API in an amorphous form has been a common approach to their formulation for several decades. Such an approach may not be suitable for poorly soluble compounds that also show poor GFA. These compounds, which demonstrate both poor solubility and poor amorphous stability, are challenging for formulation with typical polymer-based technologies due to possible phase separation and recrystallization. Ultimately, these compounds may have an increased risk of failure during pharmaceutical development, as they constitute a risk from both a bioavailability and amorphous stability perspective. In this study, we demonstrated that poor glass forming (GFA-I) APIs have increased risk of recrystallization in polymer-based amorphous solid dispersions. By contrast, mesoporous silica was shown to provide optimal stabilization for such APIs. Therefore, mesoporous silica could be an attractive formulation technology to expand the formulation toolbox for APIs that are poor glass formers. More research in the future will clarify whether mesoporous silica should become a method of choice for oral delivery of poorly soluble GFA-I compounds.

## Figures and Tables

**Table 1:** UHPLC gradient and flow rates.

Time (min)	Flow Rate (mL/min)	% (*v*/*v*) Mobile Phase A	% (*v*/*v*) Mobile Phase B
0.00	0.83	90	10
0.83	0.83	10	90
1.20	1.50	90	10
2.00	1.50	90	10
2.01	0.83	90	10

**Table 2:** Loading Capacities for Both Formulation Techniques.

Formulation	Loading Content (%) (*w*/*w*)			
	30	20	15	7.5
Haloperidol HME	Crystalline	Crystalline	Crystalline	Amorphous
Haloperidol loaded silica	Amorphous	Amorphous	Amorphous	Amorphous
Carbamazepine HME	Crystalline	Amorphous	Amorphous	Amorphous
Carbamazepine loaded silica	Amorphous	Amorphous	Amorphous	Amorphous
